# Quantification of the difference in hounsfield units of an electron density phantom between a conventional and standing computed tomography machine

**DOI:** 10.1007/s11259-025-10789-7

**Published:** 2025-06-17

**Authors:** Catherine Beck, Ashleigh V. Morrice-West, Peter Muir, Peta L. Hitchens, R. Christopher Whitton

**Affiliations:** 1 Melbourne Veterinary School, Faculty of Science, University of Melbourne, Melbourne, VIC Australia; 2 Department of Surgical Sciences, School of Veterinary Medicine, University of Wisconsin-Madison, Madison, WI USA

**Keywords:** CT machine, Hounsfield Units, Bone mineral density

## Abstract

In human CT imaging, opportunistic calculation of bone mineral density (BMD) is used to screen for osteoporosis, where BMD is estimated by Hounsfield Units (HU). There is potential to use HU for estimation of BMD in veterinary imaging. However, HU values can vary between different machines and over time. We undertook a serial measurements study to determine (1) the extent of the difference of the mean HU and noise (HU standard deviation) of eight regions of interest (ROI) of an electron density phantom scanned on two CT machines, a Siemens CT (conventional) machine and the Equina by Asto CT (standing) machine; and (2) to determine if the HU values obtained on each machine differed over time. The mean HU from 30 scans of an electron density phantom were generated from each of the two CT machines (*N* = 60; conventional and standing). Differences between machines were assessed using a linear mixed effects model. The mean HU of each positive value ROI from the conventional machine was decreased compared with the standing machine (*P* < 0.001) and the difference was greatest for the denser phantom inserts. A calibration curve equation utilising the four bone-equivalent insert plugs of the phantom, with known calcium hydroxyapatite (CaHA) concentration, was generated using linear regression to represent CaHA, and therefore BMD, for the conventional machine* (CaHA* = -29.58 + 0.98xROI_*mean*_) and the standing machine *(CaHA* = -54.53 + 0.95xROI_*mean*_). For opportunistic calculation of BMD from HU in horses, the differences in HU observed between different CT machines warrants calibration of HU using a bone equivalent phantom.

**Supplementary Information:**

The online version contains supplementary material available at 10.1007/s11259-025-10789-7.

## Introduction

In Thoroughbred racehorses, increased bone density and bone volume fraction derived from microCT is associated with proximal sesamoid (PSB) fracture (Cresswell et al. 2019; Shi et al. 2011; 2022). Similarly, increased medial PSB density obtained on conventional CT imaging has been shown to be associated with PSB fracture (Beck et al. 2025). While dual-energy x-ray absorptiometry (DXA) is the criterion standard for clinical bone mineral assessment (BMD) in humans, multiple studies have established a strong correlation between Hounsfield Unit (HU) measurements from CT imaging and BMD in humans (Schreiber et al. 2014, 2011; Pu et al. 2023; Aydin Ozturk et al. 2021; Vadera et al. 2023). This correlation suggests potential applications for BMD estimation in horses using clinical CT imaging.

BMD calculation from HU is achieved by quantitative CT (QCT) using synchronous or asynchronous scanning of a calibration phantom (Schreiber et al. 2011; Engelke et al. 2023; Lenchik et al. 2018), or through HU values of regions of interest (ROIs) obtained on routine abdominal CT imaging, so called opportunistic calculation of BMD (Schreiber et al. 2014; Pu et al. 2023; Aydin Ozturk et al. 2021; Vadera et al. 2023). In synchronous calibration, the calibration phantom is imaged in the same acquisition as the patient, whereas in asynchronous scanning the acquisition of the calibration phantom is separate to the patient (Schreiber et al. 2011; Engelke et al. 2023; Lenchik et al. 2018; Brown et al. 2017). In phantom-based QCT, HU values are converted to BMD through calibration curves using calcium hydroxyapatite (CaHA) as the reference standard (Lenchik et al. 2018; Free et al. 2018; Engelke et al. 2015). BMD calculation from CT imaging is increasingly used to screen humans for osteoporosis (Lenchik et al. 2018; Brown et al. 2017; Löffler et al. 2019; Jang et al. 2019) thus there is potential for estimation of BMD from CT imaging of horses.

When generating HU values for regions of interest (ROI), values are expressed in mean HU representing the mean HU for the ROI, and HU standard deviation within each ROI which represents noise (IAEA Human Health Series Quality Assurance Programme for Computed Tomography 2012). While actual HU values of body tissues are independent of tube current (Birnbaum et al. 2007), HU values can vary depending on the CT machine used, slice thickness, reconstruction algorithms, measurement technique, object composition, and beam energy (Lenchik et al. 2018; Free et al. 2018; Birnbaum et al. 2007; Ruder et al. 2012; Davis et al. 2018, 2017). Therefore, different CT scanners can affect HU and calibrated bone density (Free et al. 2018; Davis et al. 2018; Eggermont et al. 2018; Carpenter et al. 2014). Additionally, although advancements in CT technology have enabled more consistent and accurate HU measurements (Lenchik et al. 2018), HU values within a CT scanner may change over time (Ruder et al. 2012).

DEXA has been explored for use in horses (McClure et al. 2001) and there is a correlation between BMD values measured by QCT with those obtained by DEXA (Yamada et al. 2015). However, DEXA has not gained widespread clinical use in horses due to machine constraints and the requirement for anaesthesia. Dual energy CT may provide greater information regarding bone mineral content and bone lesions in horses. However, dual-energy CT is currently a research method rather than a clinical one (Germonpré et al. 2023).

In horses, CT density values of third metacarpal cortical, trabecular and subchondral bone are correlated with bone ash density (Waite et al. 2000; Les et al. 1994; Drum et al. 2009), where correlation is linear up to approximately 1,150 mg/ml tri-calcium phosphate (Drum et al. 2009). For PSBs, a weak correlation of CT calculated BMD with ash fraction has been established but only small sections of PSBs were used for calculation of ash fraction (Noordwijk et al. 2023). This may be explained by the variation in BMD across different regions of the PSBs (Noordwijk et al. 2023; Ayodele et al. 2021). BMD has been estimated from HU in canine studies (Decker et al. 2015; Gander Soares et al. 2022; Villamonte-Chevalier et al. 2016; Ellis et al. 2024; McCarthy et al. 2024), and with the exception of Ellis et al. (2024) and McCarthy et al. (2024), HU values were not calibrated to a CT phantom and none of the studies address the potential for variation in HU values due to the CT machine or image acquisition technical factors (Gander Soares et al. 2022; Villamonte-Chevalier et al. 2016; Ellis et al. 2024; McCarthy et al. 2024).

Therefore, the aims of this study were: 1) to determine the extent of the difference in HU values of a calibration phantom scanned with a conventional fan beam CT (cCT; Siemens CT) and a standing fan beam (sCT; Equina by Asto CT); 2) assess changes in HU values from each machine over time; and 3) to create a calibration curve for estimation of BMD for each machine. We hypothesised that there would be low variation in HU between the two machines and minimal drift in HU values for each machine over time.

## Methods

### Imaging protocol

CT scans of the phantom on two machines (cCT and sCT) were conducted between 2020 and 2023 (study period) according to clinical case presentation. For the cCT machine, the phantom was placed on the patient bed at the proximal extremity of a limb scanned as part of a post-mortem study (Beck et al. 2025) and scans were obtained between September 2020 to May 2022. For the sCT machine, the phantom was placed on the imaging pedestal and scanned separately before acquisition of a clinical scan, between October 2021 and October 2023.

### CT machines

For the cCT, imaging was undertaken on a Siemens Emotion 16 slice helical fan beam CT machine using the following image acquisition parameters: kVp: 130, mAs set to a reference of 110mAs with dose modulation employed, rotation time: 1.0 s, pitch: 0.8, field of view 230 mm, detector configuration: 16 × 0.6 mm in helical mode with an effective slice thickness of 0.75 mm, pixel spacing 0.97\0.97, image matrix 512 × 512. Images were reconstructed using a B80s sharp bone kernel with a reconstruction interval of 9.6 mm and a reconstruction diameter of 230 mm, and viewed with a window width 2000 and level 500.

For the sCT, imaging was undertaken on an Equina by Asto CT 24-slice helical fan beam CT and the following imaging parameters: 160 kVp, 8mAs, rotation time: 1 s, pitch factor: 0.55, field of view 438 × 584 mm, a helical scan velocity of 2 cm/sec; slice acquisition rate of 20 slices/sec with an effective slice thickness of 1 mm pixel spacing 0.5\0.5, image matrix 876 × 1168. Images were reconstructed using a Shepp-Logan filter within an in-plane voxel size of 0.5 mm and a slice thickness of 1.0 mm and viewed with a window width 2,000 and level 500. In February 2022, between study years 1 and 2, a 3.1 mm aluminium filter was added at the x-ray source and the software upgraded.

Over the study period both machines underwent daily calibration for all days in use and biannual technician calibration, with additional annual medical physicist calibration for the Siemens machine.

### Phantom

**Fig. 1:**
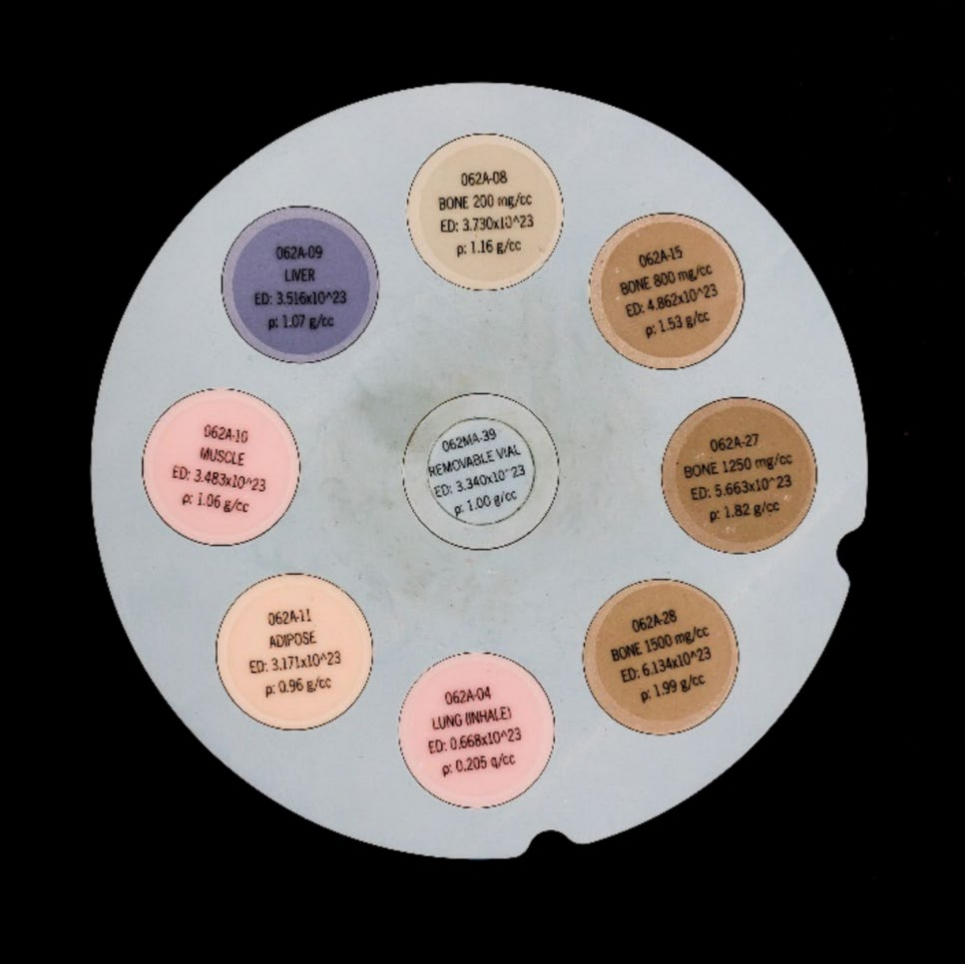
The CIRS electron density and image quality phantom system model 062 M and the eight tissue-equivalent density insert plugs (CBCT Electron Density & Image Quality Phantom, CIRS, VA, USA)

A CIRS electron density and image quality phantom system model 062 M with eight tissue-equivalent density plugs (CBCT Electron Density & Image Quality Phantom, CIRS, VA, USA) was imaged in both scanners (Fig. 1). The eight electron density inserts ranged from an electron density of 0.668 × 10^23^ electrons/cm^3^ for the lung inhale equivalent plug to 6.134 × 10^23^ electrons/cm^3^ for the dense solid bone 1,500 mg/cm^3^ calcium hydroxyapatite (CaHA) plug (Table 1).

**Table 1:** CIRS electron density and image quality phantom specifications, mean Hounsfield Unit (HU) and noise for each region of interest (ROI) obtained on the conventional and standing computed tomography machines

ROI name	Description	Physical Density g/cc	Electron Density × 10^23^ electrons/cm^3^	Relative Electron Density (relative to H_2_O)	Mean HU Conventional CT (s.d)	Mean HU Standing CT (s.d)	Noise Conventional CT (s.d)	Noise Standing CT (s.d)
1	Lung inhale equivalent	0.21	0.668	0.200	−775.36 (5.08)	−824.35 (4.57)	66.89 (5.40)	47.43 (4.20)
2	Adipose equivalent	0.96	3.171	0.949	−71.10 (2.18)	−79.09 (2.97)	76.12 (7.65)	51.39 (5.48)
3	Muscle equivalent	1.06	3.483	1.043	42.92 (2.37)	46.74 (3.42)	77.46 (6.12)	50.15 (5.33)
4	Liver equivalent	1.07	3.516	1.052	51.96 (2.30)	60.41 (3.09)	78.34 (6.11)	51.17 (6.09)
5	Trabecular bone 200 mg/cc HA	1.16	3.730	1.117	221.66 (4.73)	249.82 (3.02)	86.03 (6.20)	56.03 (6.47)
6	Solid dense bone 800 mg/cc HA	1.53	4.862	1.456	872.44 (11.04)	934.14 (11.69)	103.96 (86.85)	68.18 (8.36)
7	Solid dense bone 1,250 mg/cc HA	1.82	5.663	1.695	1,317.81 (12.41)	1,382.34 (20.77)	117.88 (9.88)	75.82 (8.48)
8	Solid dense bone 1,500 mg/cc HA	1.99	6.134	1.837	1,545.86 (11.04)	1,610.13 (22.19)	117.90 (9.09)	79.84 (8.51)

Mean HU and noise were significantly different between the standing and conventional machines for each ROI (1 to 8) (*P* < 0.001)

### Image analysis

Scans were imported into OsiriX MD (OsiriX®, version 14.0.1, Pixmeo, Switzerland). Regions of interest (ROIs) within the centre of each phantom insert were made by a board-certified radiologist (CB), mid-way along the longitudinal plane of the phantom. The ROI area was standardised at 3.02cm^2^ for each ROI and for each scan. Mean HU and noise (HU standard deviation) of each ROI for each phantom insert were obtained and exported to a custom spreadsheet.

### Statistical analysis

A serial measurement study was performed. A phantom set was defined as a singular scan of all eight ROIs within the phantom. The mean HU and noise for each of the eight ROIs within a phantom set were generated for 30 sets on the two machines (*N* = 60). Data were assessed for normality using the Shapiro–Wilk test and visual assessment of histograms. Differences in HU means and HU standard deviation for each ROI within and between the conventional and standing CT machines, and over time (years), were assessed using linear mixed effects model, adjusting for clustering on the phantom set. Coefficients and their 95% confidence intervals (95% CI) are presented. Statistical significance was set at *P* < 0.05.

Calibration curves for both CT machines were generated using the four bone-equivalent insert plugs of the phantom, represented by ROIs 5–8, each with a known CaHA concentration (200, 800, 1,250 and 1,500 mg/cm^3^ CaHA). For each machine, the calibration curve was defined as a linear equation, with the slope and y-intercept obtained via linear regression. The calibration curve was represented by Y = β_0_+ β_1_${ROI}_{mean}$ where β_0_ is the constant and β_1_ is the slope coefficient generated from the regression model (Free et al. 2018). The outcome variable was CaHA concentration predicted by mean HU.

Statistical analysis was performed using Stata/MP 18.0 (StataCorp Stata Statistical Software: Release 18, StataCorp LLC, College Station, Texas USA).

## Results

CIRS electron density and image quality phantom specifications, mean HU and noise obtained on the conventional and standing CT machines are reported in Table 1. The results of the mixed-effects model analysing the differences in mean HU and HU standard deviation between the conventional and standing CT machines across eight different ROIs are presented in Supplementary File 1.

The mean HU of each ROI with a positive value was lower for the cCT machine than for the sCT machine. For the negative values ROIs, ROIs 1 and 2, mean HU values were lower (more negative) for the sCT compared with the cCT. (*P* < 0.001; Table 1 and Supplementary File, Figure S1, Table S1, Figure S2). For one phantom set obtained with the sCT machine, the mean HU value was an outlier for ROIs 2, 3 and 4 (Supplementary File, Figure S1). The difference between the machines remained for all ROIs after removal of this outlier (*P* < 0.001). For the cCT machine, mean HU for the lowest bone equivalent ROI (ROI 5; 221.66 HU) was 28.16 HU lower than the sCT (249.82 HU). The greatest difference was for ROIs 6–8 representing the denser phantom inserts (range 4.862–6.134 × 10^23^ electrons/cm^3^) and lowest for the mid-range ROIs 2–4**.** For the denser bone equivalent inserts of ROIs 6–8, the mean HU values for the cCT ranged from 61.61–64.53 HU lower than the sCT (Table 1).

Noise was lower for the sCT machine than the cCT machine (*P* < 0.001; Table 1, Figure S3, Table S2, Figure S4). For both machines, noise was greatest for ROIs 7 and 8 representing the densest phantom inserts (Table 1).

The mean HU for the ROI of each phantom insert obtained over three years are provided in Table S3. For the cCT machine, a difference was detected for ROI4 (*P* = 0.029), no difference in ROI mean HU for all other ROIs was detected. For the sCT machine, there was an increase in mean HU for ROIs 5–8 (*P* < 0.001) over the three-year study period, for ROIs 1–4, a difference was not detected.

**Fig. 2:**
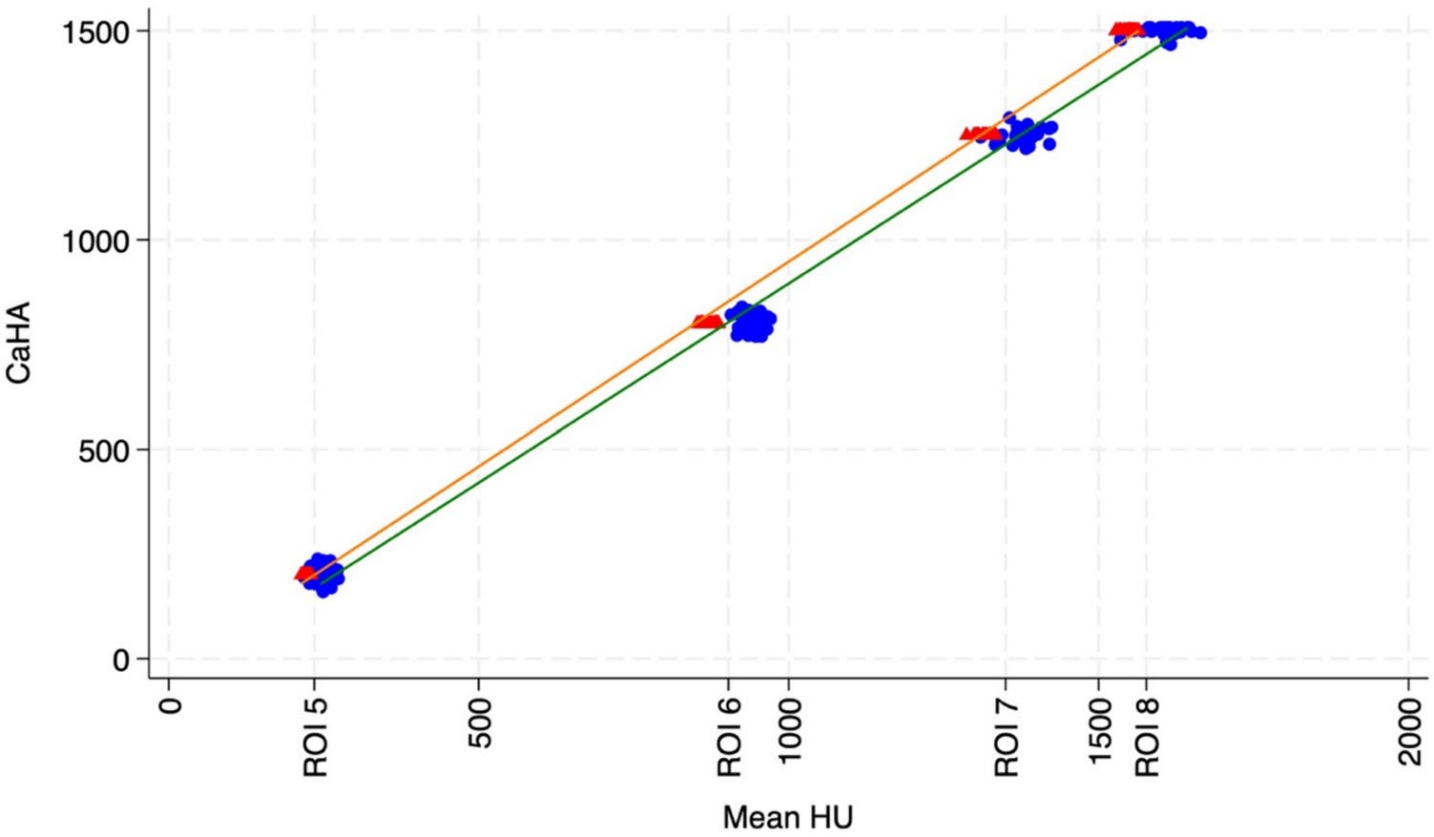
Calibration curve representing the linear regression line of best fit of mean HU obtained from the ROIs of the four bone-equivalent phantom inserts 5 to 8 against the known CaHA mg/cm.^3^ concentration of each insert for the conventional (orange) and standing CT (green). The calibration curve equations are Conventional Machine: ${CaHA}_{cCT}=-29.58+0.98\times {ROI}_{mean}$ Standing Machine: ${CaHA}_{sCT}=-54.53+0.95\times {ROI}_{mean}$. Jitter was applied to the data points to prevent overlap (degree 4; *N* = 60)

For asynchronous calculation of CaHA values from HU, a calibration curve was generated using linear regression line of best (Fig. 2). The calibration curve equation is represented as Eq. (1) for the cCT:

1 $${CaHA}_{cCT}=-29.58+0.98\times {ROI}_{mean}$$

and Eq. (2) for the sCT machine:

2 $${CaHA}_{sCT}=-54.53+0.95\times {ROI}_{mean}$$

The sCT machine produced greater mean HU for each ROI (*P* < 0.001), with the difference between machines for the mean HU increasing with greater density inserts of the phantom due to the slightly steeper slope coefficient (Fig. 2).

## Discussion

Our results indicate a difference in HU values obtained on a conventional CT and a standing CT machine. The difference varied with the ROI density, with the greatest difference being for phantom plug inserts representing a density range of 200–1,500 mg/cm^3^ CaHA. For all ROIs with a positive HU value, the conventional machine produced lower HU values than the sCT machine. There was a small upward trend of HU values for the soft tissue value ROIs obtained on the cCT and for the denser ROIs imaged on the sCT over the three-year period.

The greatest interscanner differences were observed for the higher density inserts, consistent with previous investigations (Birnbaum et al. 2007; Ruder et al. 2012). Whilst these findings are translatable to clinical imaging, beam hardening and scatter from the interaction of x-rays with the patient can impact HU measurements (Sande et al. 2010). Consequently, the actual HU obtained during clinical scanning may vary. With the sCT approach, both limbs of the horse are scanned in the same field of view. Consequently, beam hardening and scatter are likely to be greater with this system than with the conventional or other CT systems where the distal limbs are often scanned separately (Mageed 2022).

The mean HU values for positive value ROIs on conventional cCT machine were lower than for the sCT machine, and for the two negative value ROIs, the standing CT mean HU was lower. There was a difference in scanning parameters for each machine which likely contributed to the differences in observed variations in HU values. For the conventional CT, images were acquired using a lower kVp and higher mAs, than the standing CT machine. The HU value for a given material is energy dependent. Interscanner differences diminish as kVp increases from 80–140 (Sande et al. 2010). In our study, a kVp difference of 30 was present between the machines which may partially explain the difference in HU values obtained. The field of view (FOV) for the cCT machine was smaller than the sCT machine. The smaller FOV reduces the amount of scatter radiation produced, partial volume averaging and beam hardening artefacts. Therefore, differences in FOV may also explain the differences in HU obtained (Bushberg et al. 2021a). Another explanation may be image reconstruction, as proprietary reconstruction was used by the software running each machine. The image reconstruction kernel has an inconsistent effect on tissue attenuation depending on the scanner (Free et al. 2018; Birnbaum et al. 2007; Diwakar and Kumar 2018).

Tube current has been shown to have no significant effect on HU values (Birnbaum et al. 2007; Davis et al. 1076), so the difference in tube current between the two machines may not explain the observed HU differences. Tube current does influence image noise with a higher current leading to decreased noise (IAEA Human Health Series Quality Assurance Programme for Computed Tomography 2012; Diwakar and Kumar 2018). Interestingly, in our study, noise was greatest on the cCT images acquired with the higher tube current. This finding may be explained by the lower tube voltage used by the cCT machine as decreasing kVp increases image noise (Diwakar and Kumar 2018). Slice thickness also affects noise, with thicker slices having reduced image noise (Diwakar and Kumar 2018). Whilst slice thickness was similar for both machines it was slightly less for the cCT machine. It is therefore likely that the greater noise observed in the cCT images is a result of a combination of tube voltage and slice thickness, in addition to other factors such as the reconstruction algorithm.

The mean HU values for the cCT machine did not differ across the three-year study period for all ROIs except ROI4. For both ROIs in the soft tissue density range, ROIs 3 and 4, there was a small upward trend for the mean HU of both ROIs with ROI 4 reaching statistical significance. The observed increase in mean HU for ROI4 was 2.59 HU over the three years which is not likely to be clinically significant. However, this highlights the importance of regular machine calibration. For the sCT, mean HU did not differ for the lower density ROIs. However, there was a difference for the denser ROIs on the sCT machine. This difference may be due to the addition of an aluminium filter and software upgrade between study years 1 and 2 with this machine. Aluminium filters remove lower energy x-rays from the beam, reducing patient dose and increasing the mean energy of the x-ray beam, known as beam hardening (Bushberg et al. 2021b). Beam hardening may lead to differences in HU values for higher density inserts due to the greater number of photoelectric interactions which are energy dependant. This is in contrast to the lower density inserts where the less energy dependent Compton scattering predominates (Bushberg et al. 2021b).

Mean HU values obtained from asynchronous scanning of a calibration phantom were used to generate a calibration curve for each machine in the study and these curves can be used for BMD estimation. The use of a calibration phantom scanned synchronously has been described in QCT imaging of cadaver limbs in the horse (Les et al. 1994; Olive et al. 2010a, b; Liley et al. 2018; Young et al. 2007). However, placement of a BMD calibration phantom in the field of view for synchronous calibration when scanning standing horses is not feasible with most systems. When utilising a calibration phantom, the scan acquisition must be performed with the same parameters as the patient scan to minimise errors induced by factors such as the reconstruction algorithm. Additionally scans of the phantom should be obtained on the same day as the patient after daily machine calibration.

This study was undertaken using two different fan-beam CT machines and as such the results are not translatable to cone-beam CT machines. Whilst cone-beam CT produces better spatial resolution than fan-beam CT, fan-beam CT has fewer artifacts, particularly motion artefact, less noise, greater signal-to-noise ratio, and better soft tissue resolution than cone-beam CT (Lechuga et al. 2016). Additionally HU values cannot be calculated directly from cone-beam CT but must be derived from grey scale levels (Lechuga et al. 2016; Reeves et al. 2014). The beam hardening artifact seen with cone-beam CT increases image noise leading to less accurate density values (Lechuga et al. 2016). Therefore, fan-beam CT systems are preferred for opportunistic estimation of BMD in horses.

This study has several limitations. The sCT underwent a software upgrade between the first and second years of scanning which may have influenced the results obtained from this machine. A limitation of this study was the use of the commercially validated CIRS CBCT electron density phantom rather than an American College of Radiology (ACR) CT accredited phantom which may restrict comparisons with studies utilising ACR CT accredited phantoms. The CIRS phantom was selected due to its wide range of mineral densities that encompass the wide range of bone mineral densities observed in horses, which may exceed those available in standard calibration phantoms. Additionally, the image acquisition process for the phantom was different for each machine. For the conventional machine the phantom was placed on the CT table, at the extremity of the limb of a horse and scanned asynchronously as part of the post-mortem CT program.^4^ For the sCT, the phantom acquisition was performed as a separate scan before scanning the patient, and the difference in position of the phantom may have an effect on calculated HU (Free et al. 2018). With the sCT machine, there was one phantom set which had outlier values for ROIs 2–4. These may have been due to differences in the position of the phantom on the imaging pedestal as tilting the position of the phantom in the field of view may affect the HU values obtained (Free et al. 2018).

## Conclusions

Our results confirm that HU obtained from two CT systems vary. Whilst the difference is small, the difference in HU obtained on different machines should be considered when using HU for discrimination of disease, and for comparisons of values between machines. For estimation of BMD with CT scanning of horses, calibration curves obtained through linear regression of bone equivalent CT phantoms should be used to generate calibration curves. Further work is required to establish the correlation of CT-derived BMD values in horses, and other methods for establishing BMD such as DXA, microCT, and ash fraction.

## Supplementary Information

Below is the link to the electronic supplementary material.

Supplementary file1 (DOCX 404 KB)

## Data Availability

The data sets generated during and analysed during the current study are available at: https://figshare.unimelb.edu.au/articles/dataset/CT_machine_comparison/28426541?file=52394153.
