# Supplementary material for: Quantification of the difference in hounsfield units of an electron density phantom between a conventional and standing computed tomography machine
Source: Vet Res Commun. 2025 Jun 17;49(4):228. doi: 10.1007/s11259-025-10789-7 (PMC12174245; doi:10.1007/s11259-025-10789-7)
Supplement: Supplementary file 1 — Supplementary file1 (DOCX 404 KB) [file 11259_2025_10789_MOESM1_ESM.docx]

**Supplementary item 1**


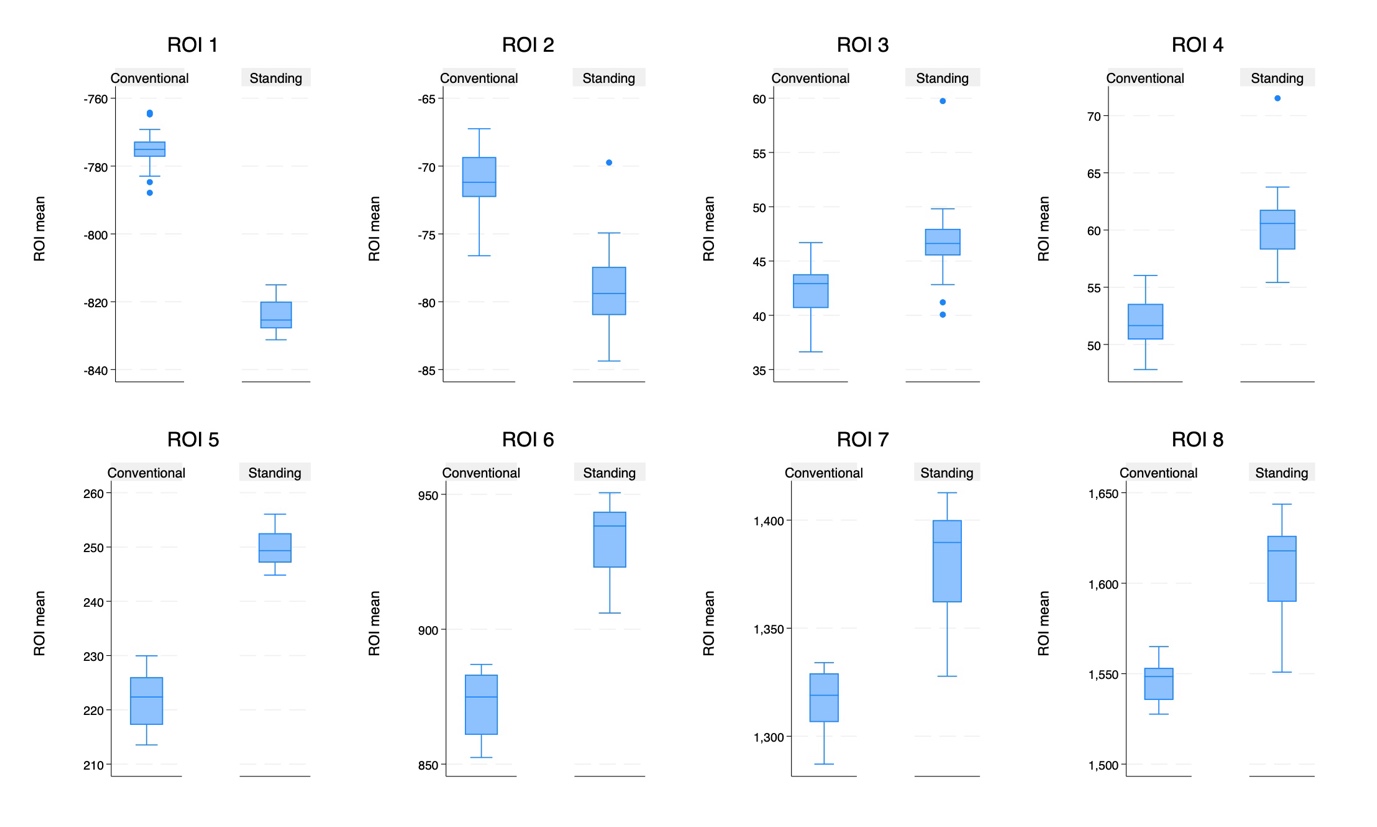


**Fig S1**: Box plot of the mean Hounsfield Unit (HU) calculated from the conventional and standing computed tomography machines. The mean HU for the conventional machine for the majority of densities (ROI 3-8) were lower than for the standing machine (P < 0.001) across the regions of interest with positive mean HU. For ROIs 1 and 2, with negative mean HU, values were lower (more negative) for the standing CT compared with the conventional CT. For the conventional machine, on average the mean HU was 49 HU lower across all regions-of-interest (ROIs). However, the difference varied across the phantom sets. The greater value outliers for ROIs 2,3 and 4 on the standing machine are from the same phantom set

**Table S1** Linear mixed-effects model results of mean Hounsfield Unit (HU) for each region-of-interest (ROI) for the standing computed tomography (CT) and the conventional CT machines over three years. Mean HU for the conventional machine is lower than for the standing machine for all ROIs (P < 0.001).

| Parameter | Coefficient | Std. Error | z-value | P-value | 95% CI Lower |
| --- | --- | --- | --- | --- | --- |
| Machine |  |  |  |  |  |
| Standing | Reference |  |  |  |  |
| Conventional | -49.09 | 2.52 | -19.45 | <0.001 | -54.03, -44.14 |
| **ROI** |  |  |  |  |  |
| ROI 1 | Reference |  |  |  |  |
| ROI 2 | 704.25 | 2.29 | 307.81 | <0.001 | 699.77, 708.74 |
| ROI 3 | 817.78 | 2.29 | 357.43 | <0.001 | 813.29, 822.26 |
| ROI 4 | 827.32 | 2.29 | 361.60 | <0.001 | 822.83, 831.80 |
| ROI 5 | 997.02 | 2.29 | 435.77 | <0.001 | 992.54, 1001.51 |
| ROI 6 | 1647.79 | 2.29 | 720.21 | <0.001 | 1643.31, 1652.28 |
| ROI 7 | 2093.16 | 2.29 | 914.87 | <0.001 | 2088.68, 2097.65 |
| ROI 8 | 2321.21 | 2.29 | 1014.55 | <0.001 | 2316.73, 2325.70 |
| **Machine × ROI Interaction** |  |  |  |  |  |
| Machine × ROI 2 | 41.00 | 3.24 | 12.67 | <0.001 | 34.66, 47.35 |
| Machine × ROI 3 | 53.32 | 3.24 | 16.48 | <0.001 | 46.98, 59.66 |
| Machine × ROI 4 | 57.44 | 3.24 | 17.75 | <0.001 | 51.10, 63.78 |
| Machine × ROI 5 | 77.15 | 3.24 | 23.84 | <0.001 | 70.81, 83.49 |
| Machine × ROI 6 | 110.70 | 3.24 | 34.21 | <0.001 | 104.36, 117.04 |
| Machine × ROI 7 | 113.53 | 3.24 | 35.09 | <0.001 | 107.19, 119.87 |
| Machine × ROI 8 | 113.26 | 3.24 | 35.00 | <0.001 | 106.92, 119.60 |
| Intercept | -775.36 | 1.78 | -435.21 | <0.001 | -778.85, -771.86 |


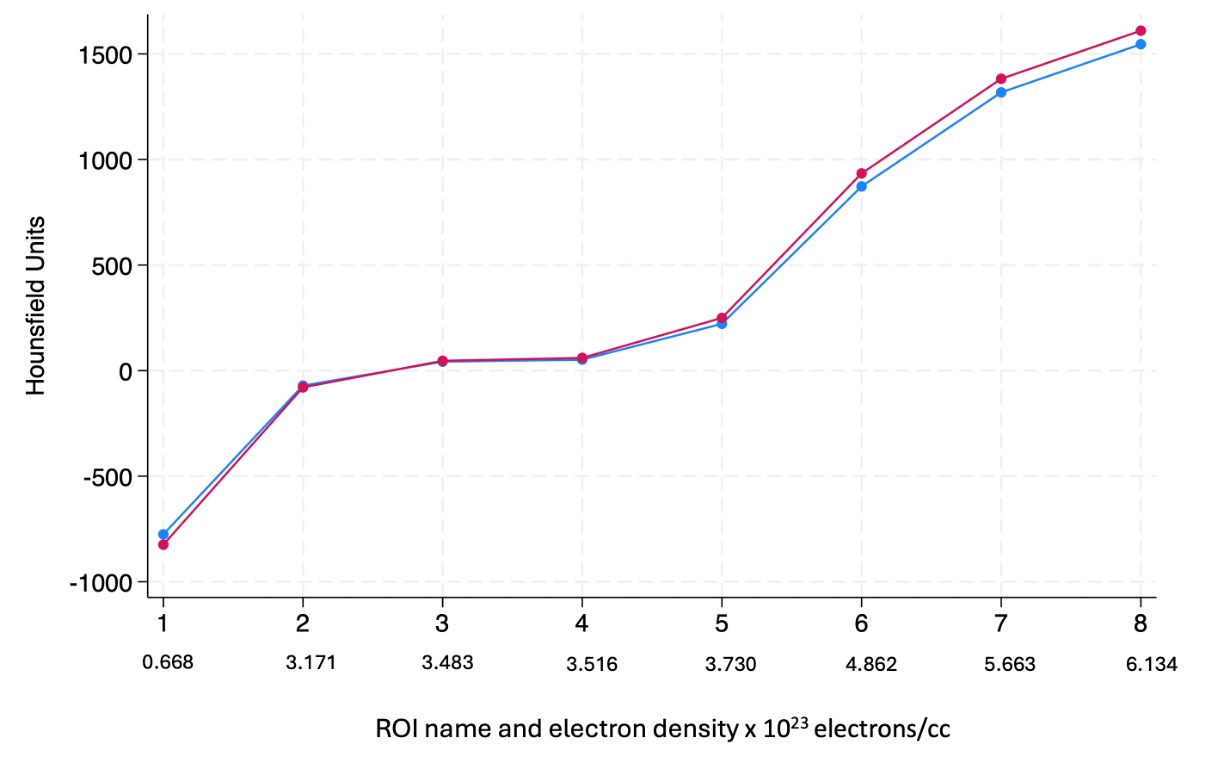


**Fig S2** Mixed-effects model plot of the marginal mean Hounsfield Units (HU) for each region-of-interest (ROI) for the conventional (blue line) and standing (red line) computed tomography machines. The mean HU for each ROI is lower for the standing machine than the conventional machine for the negative value ROIs 1 and 2 and higher for the positive value ROIS 3-8. As electron density increases the difference in ROI mean HU increases


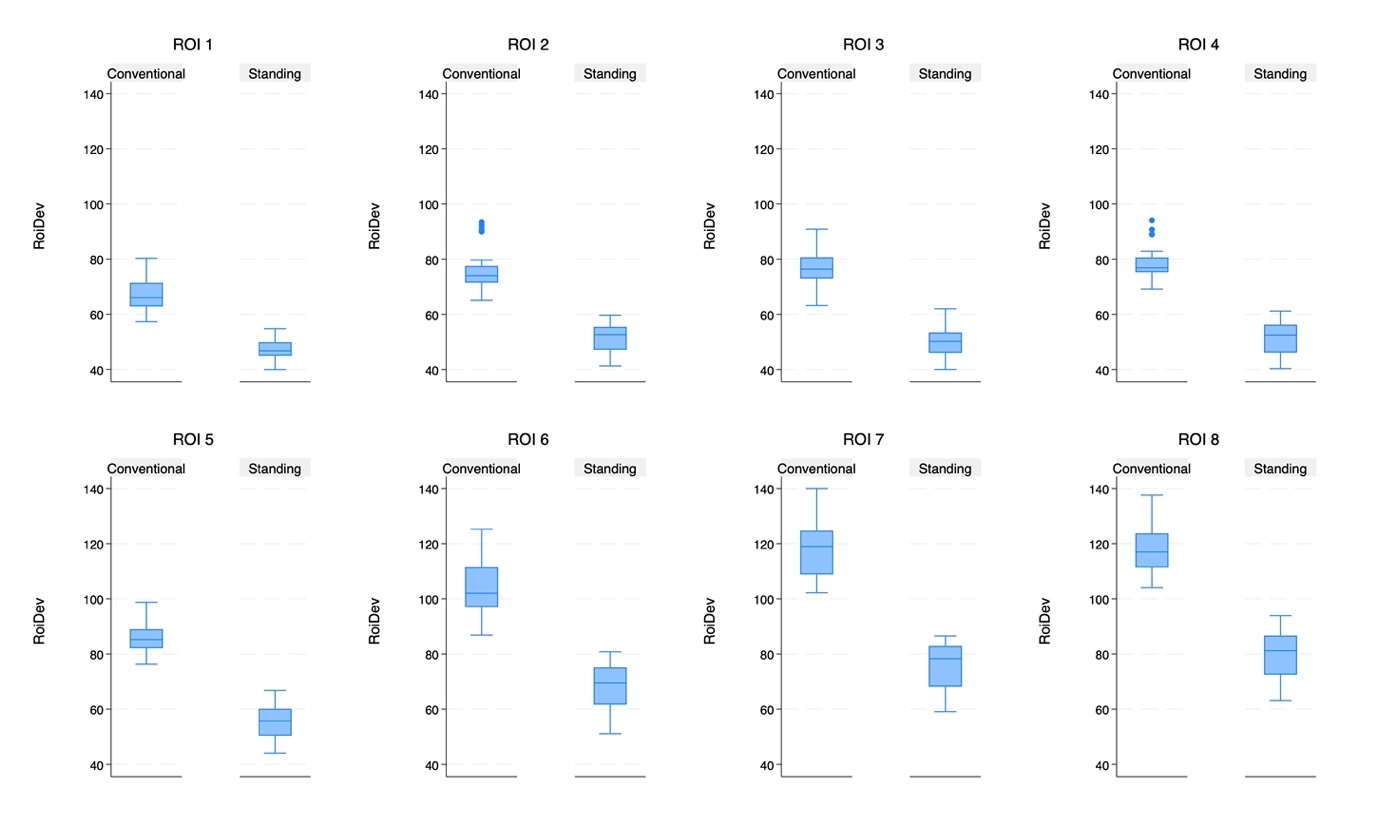


**Fig S3** Box Plot of the noise (Hounsfield Unit [HU] standard deviation) calculated from the conventional and standing computed tomography machines. Noise is greatest for the conventional machine and for inserts 7 and 8

**Table S2**. Mixed-effects model results of noise (Hounsfield Unit [HU] standard deviation) for each region-of-interest (ROI) for the conventional CT and standing CT machines over 3 years.

The standing machine produces less noise than the conventional machine (P < 0.001) by an average of about 19.46 HU. Noise increased across ROI and was greatest for phantom inserts 7 and 8.

| Parameter | Coefficient | Std. Error | z-value | P-value | 95% CI |
| --- | --- | --- | --- | --- | --- |
| Machine |  |  |  |  |  |
| Standing | Reference |  |  |  |  |
| Conventional | -19.46 | 1.85 | -10.54 | <0.001 | -23.08, -15.84 |
| **ROI** |  |  |  |  |  |
| **ROI 1** | Reference |  |  |  |  |
| ROI 2 | 9.23 | 1.23 | 7.48 | <0.001 | 6.81, 11.65 |
| ROI 3 | 10.57 | 1.23 | 8.56 | <0.001 | 8.15, 12.99 |
| ROI 4 | 11.45 | 1.23 | 9.28 | <0.001 | 9.03, 13.87 |
| ROI 5 | 19.15 | 1.23 | 15.51 | <0.001 | 16.73, 21.57 |
| ROI 6 | 37.08 | 1.23 | 30.03 | <0.001 | 34.66, 39.50 |
| ROI 7 | 50.99 | 1.23 | 41.30 | <0.001 | 48.57, 53.41 |
| ROI 8 | 51.01 | 1.23 | 41.32 | <0.001 | 48.59, 53.43 |
| **Machine × ROI Interaction** |  |  |  |  |  |
| Machine × ROI 2 | -5.27 | 1.75 | -3.02 | 0.003 | -8.69, -1.85 |
| Machine × ROI 3 | -7.85 | 1.75 | -4.50 | <0.001 | -11.27, -4.43 |
| Machine × ROI 4 | -7.71 | 1.75 | -4.41 | <0.001 | -11.13, -4.29 |
| Machine × ROI 5 | -10.54 | 1.75 | -6.04 | <0.001 | -13.96, -7.12 |
| Machine × ROI 6 | -16.33 | 1.75 | -9.35 | <0.001 | -19.75, -12.91 |
| Machine × ROI 7 | -22.60 | 1.75 | -12.94 | <0.001 | -26.02, -19.18 |
| Machine × ROI 8 | -18.60 | 1.75 | -10.65 | <0.001 | -22.03, -15.18 |
| Intercept | 66.89 | 1.31 | 51.24 | <0.001 | 64.33, 69.44 |


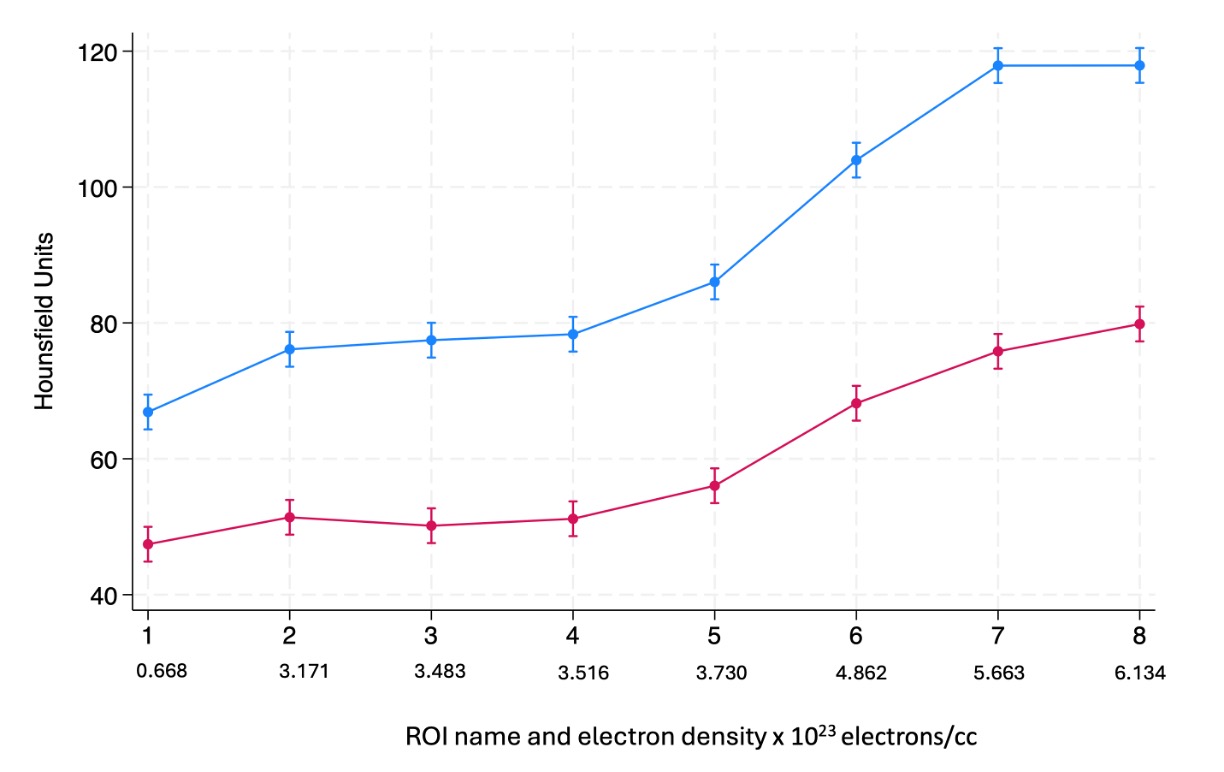


**Fig S4** Mixed-effects model plot of noise (Hounsfield Unit [HU] standard deviation) for each region-of-interest (ROI) for the conventional (blue line) and standing (red line) computed tomography machines. The noise for each ROI is lower for the standing machine than the conventional machine. For both machines noise increased across ROI and was greatest for phantom inserts 7 and 8

**Table S3**: Mean Hounsfield units (HU) for eight regions-of-interest (ROI) conducted over 30 phantom sets scanned on the conventional CT and standing CT machines over three years (N=60). The phantom was scanned on the conventional CT machine in 2020, 2021 and 2022 and on the standing CT machine in 2021, 2022 and 2023.

| Conventional | | Year 1 | Year 2 | Year 3 | P-value |
| --- | --- | --- | --- | --- | --- |
| 1 | | -775.21 (6.41) | -775.63 (4.46) | -774.91 (5.87) | 0.886 |
| 2 | | -72.49 (2.40) | -70.89 (2.23) | -70.48 (1.67) | 0.080 |
| 3 | | 41.58 (2.23) | 42.24 (2.64) | 43.41 (1.73) | 0.121 |
| 4 | | 50.15 (2.06) | 52.25 (2.34) | 52.74 (1.85) | 0.029 |
| 5 | | 219.94 (4.12) | 221.32 (4.81) | 223.64 (4.89) | 0.117 |
| 6 | | 870.46 (9.07) | 872.17 (10.97) | 874.44 (13.43) | 0.487 |
| 7 | | 1319.82 (12.06) | 1317.80 (11.00) | 1316.32 (16.39) | 0.596 |
| 8 | | 1550.83 (11.96) | 1546.37 (10.87) | 1541.10 (10.09) | 0.078 |
| Standing | Year 1 | Year 2 | Year 3 | P-value | |
| 1 | -822.83 (4.20) | -823.72 (5.42) | -826.50 (3.45) | 0.052 | |
| 2 | -77.41 (3.29) | -80.86 (2.59) | -79.01 (2.09) | 0.210 | |
| 3 | 48.26 (4.18) | 44.19 (2.66) | 47.78 (1.48) | 0.747 | |
| 4 | 61.86 (3.74) | 58.24 (2.11) | 61.13 (2.07) | 0.589 | |
| 5 | 247.30 (2.33) | 250.46 (2.69) | 251.71 (2.27) | < 0.001* | |
| 6 | 919.80 (5.13) | 939.81 (6.76) | 942.82 (4.55) | < 0.001* | |
| 7 | 1357.22 (11.16) | 1390.33 (10.44) | 1399.47 (7.68) | < 0.001* | |
| 8 | 1584.03 (12.30) | 1618.75 (12.33) | 1627.60 (10.27) | < 0.001* | |
